# Contrasting EfficientNet, ViT, and gMLP for COVID-19 Detection in Ultrasound Imagery

**DOI:** 10.3390/jpm12101707

**Published:** 2022-10-12

**Authors:** Mohamad Mahmoud Al Rahhal, Yakoub Bazi, Rami M. Jomaa, Mansour Zuair, Farid Melgani

**Affiliations:** 1 Applied Computer Science Department, College of Applied Computer Science, King Saud University, Riyadh 11543, Saudi Arabia; 2 Computer Engineering Department, College of Computer and Information Sciences, King Saud University, Riyadh 11543, Saudi Arabia; 3 Computer Science Department, College of Computer and Information Sciences, King Saud University, Riyadh 11543, Saudi Arabia; 4 Department of Information Engineering and Computer Science, University of Trento, 38123 Trento, Italy

**Keywords:** coronavirus, classification, deep learning, transfer learning, ultrasound images

## Abstract

A timely diagnosis of coronavirus is critical in order to control the spread of the virus. To aid in this, we propose in this paper a deep learning-based approach for detecting coronavirus patients using ultrasound imagery. We propose to exploit the transfer learning of a EfficientNet model pre-trained on the ImageNet dataset for the classification of ultrasound images of suspected patients. In particular, we contrast the results of EfficentNet-B2 with the results of ViT and gMLP. Then, we show the results of the three models by learning from scratch, i.e., without transfer learning. We view the detection problem from a multiclass classification perspective by classifying images as COVID-19, pneumonia, and normal. In the experiments, we evaluated the models on a publically available ultrasound dataset. This dataset consists of 261 recordings (202 videos + 59 images) belonging to 216 distinct patients. The best results were obtained using EfficientNet-B2 with transfer learning. In particular, we obtained precision, recall, and *F*1 scores of 95.84%, 99.88%, and 24 97.41%, respectively, for detecting the COVID-19 class. EfficientNet-B2 with transfer learning presented an overall accuracy of 96.79%, outperforming gMLP and ViT, which achieved accuracies of 93.03% and 92.82%, respectively.

## 1. Introduction

COVID-19 is an infectious disease caused by a virus from the coronavirus strain. Coronavirus is a phrase derived from the Latin word corona, which means crown. The World Health Organization (WHO) reports that the number of those infected with this virus is rising quickly. According to statistics released on 12 March 2021, more than 116 million instances and around 2.5 million deaths had already been confirmed. [1]. The most well-known symptoms of COVID-19 are fever, fatigue, and dry cough; other less-common symptoms can include pain, nasal congestion, and loss of taste and smell. The risk of serious complications is more significant among the elderly and people with health problems. 

In addition to the ordinary real-time polymerase chain reaction (RT-PCR) test, medical images are progressively used for screening and monitoring the disease. Indeed, medical imaging such as ultrasound, computed tomography (CT), and X-ray are important elements in medical practice as they allow scientists to learn more about the disease in a noninvasive manner. Furthermore, the automatic analysis of these images using machine learning methods can greatly assist in monitoring the effectiveness of the treatment and adjusting protocols based on its severity. More recently, ultrasound imaging has also been used for disease screening as it has many advantages in terms of being relatively cost effective compared to other imaging techniques such as CT, being safe, and providing efficient and immediate healthcare information [2].

Deep learning techniques have recently demonstrated their dominance over traditional approaches in numerous domains, such as computer vision and image classification [3,4]. These techniques have achieved encouraging results in classifying medical images [5,6]. Several research works have applied deep learning networks to detect COVID-19 using lung ultrasound (LUS) [7,8,9,10,11,12,13], CT scan [14,15,16,17,18,19,20,21,22], or CXR images [23,24,25,26,27,28,29,30,31]. The development of an efficient and accurate system for detecting COVID-19 is still challenging, and the need for detecting COVID-19 cases as soon as possible with high accuracy could save the rest of the community from this pandemic. Furthermore, the appearance of new variants of SARS-CoV-2 is encouraging researchers to develop and improve new systems that are able to detect patients infected by new variants. 

The objective of this work is to design an automatic system for detecting COVID-19 using LUS. To this end, we employ several deep learning models. In the proposed models, we explore two learning approaches: transfer learning and learning from scratch. The former approach, transfer learning, is used in the case of a relatively limited number of training samples; therefore, we fine-tune a pre-trained model instead of training a new model from scratch as in the later approach. More specifically, we develop three well-known models—EfficientNet-B2 [32], gMLP [33], and ViT [34]—for classifying LUS images. We evaluate the efficiency of these models within a multiclass problem where the goal is to classify LUS images into COVID-19, pneumonia, or normal classes.

The main contributions of this work are as follows:

- We propose a deep learning-based model for the automatic detection of COVID-19 using LUS images, to increase the accuracy and the speed of detecting COVID-19 compared with the routine rRT-PCR test.
- Three deep learning models are proposed and evaluated: EfficientNet-B2, gMLP, and ViT.
- We explore the effectiveness of the proposed models in two learning approaches: transfer learning and learning from scratch.

The remainder of this article is organized as follows: Section 2 lists the main related works. In Section 3, we present a detailed description of the proposed deep learning models. Section 4 is dedicated to describing the dataset and presenting the experimental results obtained with the proposed approach and discussing our findings. Finally, Section 5 presents conclusions and future works.

## 2. Related Works

The literature on COVID-19 reports several methods for the analysis of medical images such as of CT images [35,36,37,38,39,40,41], X-ray [42,43,44,45,46,47], and LUS [7,8,9,10]. For instance, Silva et al. [35] proposed a voting-based approach, where the images from a given patient are classified using a group in a voting system. In [36], the authors implemented a bidirectional classification system using differential evolution algorithms. In [37], the authors proposed a contrastive learning method for jointly learning on heterogonous datasets. In [38], the authors proposed a multiscale feature fusion method for enhancing the detection accuracy. In [39], Zhou et al. proposed a method that allows the segmentation and identification of the infected region images from different sources. In another work [40], a method based on an adaptive feature selection deep guided forest method was proposed. 

Similarly other approaches have been developed for disease detection using X-ray imagery [42,43,44,45,46,47]. For example, in [42], the authors developed a model for classifying images into three different classes: non-COVID, pneumonia, and COVID. The authors in [43] used different pre-trained Convolutional Neural Network (CNN) architectures for feature generation and investigated different classifiers to classify the extracted features. They found that the best results were obtained using MobileNet as a pre-trained CNN combined with a Support Vector Machine (SVM) classifier. The authors in [44] proposed a transfer learning approach based on decomposition techniques to detect the class boundaries. In [45], the authors proposed a capsule network composed of four convolutional layers and three capsule layers for handling class imbalance problems. In [46], the authors proposed a COVID data-based network that combines segmentation and data augmentation in order to improve the detection accuracy. In [47], the authors suggested employing a bilateral low-pass filter and a histogram equalization technique to pre-process the images. Then, a pseudo-color image created from the original and filtered images is given progressively to a CNN model for classification.

In the context of detecting COVID-19 using LUS, the authors in [9] suggested a spatial transformer network that simultaneously predicts the severity of the disease and provides weakly supervised localization of pathological artifacts. They also presented a technique for frame score aggregation at the video-level based on uninorms. Their proposed model achieved an *F*1 score of 71.4% in frame-based classification. The authors of [13] applied pre-trained residual CNN models (ResNet18/ResNet50). They evaluated their proposed models using a dataset with four to seven classes; their proposed models achieved good results, with an *F*1 score of 98%. The author of [10] proposed a light deep learning model for detecting COVID-19 using ultrasound images. Their model achieved very good results in terms of the training time, but with a low overall accuracy of 83%. 

The literature shows that it is indeed possible to develop a deep learning system for the automatic detection of COVID-19 using LUS, to improve the performance of the systems in terms of classification accuracy, which encourages us to present this study. 

## 3. Materials and Methods

### 3.1. Dataset Description 

In the experiments, we used the ultrasound dataset proposed in [8,11]. This dataset consists of 255 LUS recordings (196 videos and 59 images) belonging to 216 distinct patients. This dataset includes samples from COVID-19, bacterial pneumonia, and healthy patients, as indicated in Table 1 [8]. 

The dataset was collected from different sources, including clinical information provided voluntarily by hospitals or ultrasound instructors at academic institutions, LUS recordings published in other scholarly works, community platforms, open medical repositories, and health-tech firms. The data were acquired using a variety of ultrasound devices with linear and convex probes. Because of their higher frequency, linear probes have a higher resolution, which makes it easier to analyze problems along the pleural line [48]. The linear probe does not analyze deeper lung tissue because it penetrates the tissue less than the convex probe, which can interfere with the distinction of B-lines [49].

**Figure 1:**
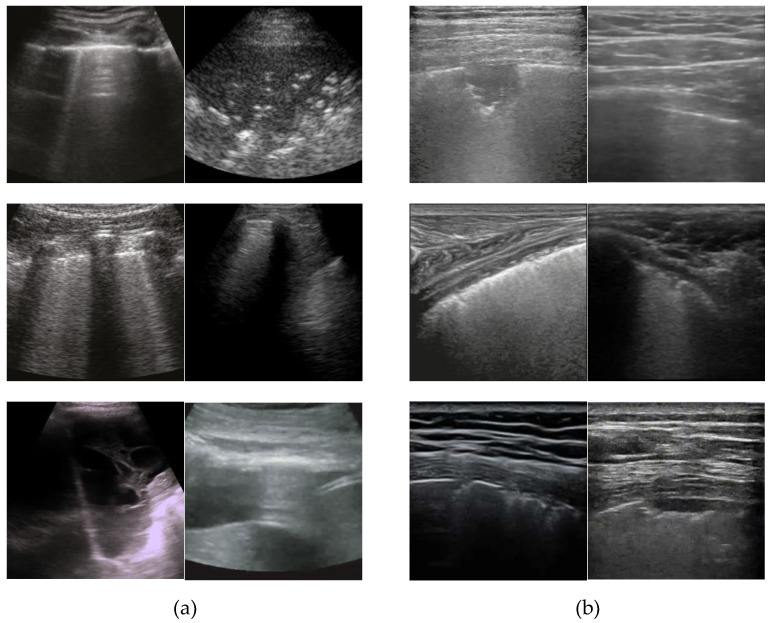
Examples of ultrasound images obtained from different probes and records using (**a**) a convex sensor and (**b**) a linear sensor.

Figure 1 shows examples of ultrasound images obtained from different probes and records, where the left two columns (a) represent images from several records captured using convex sensor, and the right two columns (b) represent images from different records captured using a linear sensor.

### 3.2. Compound Scaling Network

The recent trend of image classification problems is training deep neural network architectures, mainly CNN, to predict the labels of test images. A CNN network is a cascade of several convolution layers that have trainable weights and biases. Each layer performs a convolution operation on the input data followed by optional nonlinear operation. Following the success of AlexNet [50] on the ImageNet Large Scale Visual Recognition Challenge (ILSVRC), CNNs have been successfully applied in various research domains, including medical image analysis. The performance of ConvNets has been significantly improved by training deeper architectures (network architectures with several convolution layers arranged in various ways) such as GoogleNet [51], ResNet [52], and more recently, GPipe [53]. These architectures perform scaling of the ConvNets by increasing the number of layers (depth increase), number of channels in each layer (width increase), or image resolution. EfficientNet [32] is currently the only model that performs scaling in all of the three dimensions in a principled way.

**Figure 2:**
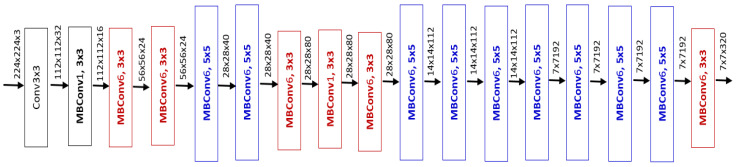
The EfficientNet-B2 Architecture. The numeric value next to MBConv indicates a multiplication factor for the input channels. For example, MBConv6 means the output channel size is 6 times the input channel size. Labels in the form of a × b represent kernel size [32].

The authors of EfficientNet show that although scaling ConvNets in one of the three dimensions (depth, width, and image resolution) improves performance, the gain saturates quickly as the network becomes bigger. In order to overcome this, they proposed a compound scaling method that uniformly scales network depth, width, and resolution using fixed scaling coefficients. Moreover, they validated the importance of balancing a network in all dimensions first by developing a mobile-size baseline network called EfficientNet-B0. Then, starting from this baseline network, they applied the proposed scaling method to obtain eight variants of the EfficientNet model. Figure 2 shows the architecture of EfficientNet-B2. The proposed models significantly outperform other ConvNet architectures on the ImageNet classification problem while having fewer parameters and running much faster during inference, an important property for real-time applications such as the one considered in this work. In addition, the features learned by the networks are transferable and achieve impressive results on a wide range of datasets.

**Figure 3:**
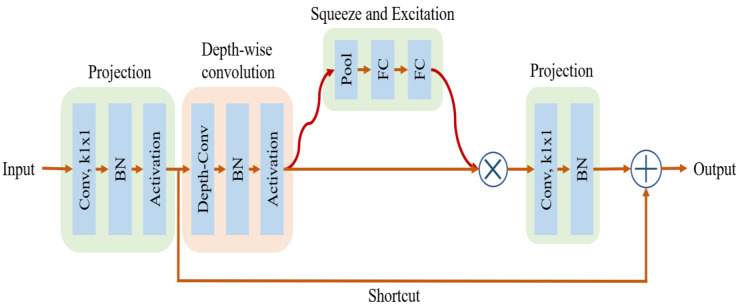
A mobile inverted bottleneck layer used for the EfficientNet family of models. (BN—Batch normalization layer; FC—Fully connected layer).

The baseline network (EfficientNet-B0) uses the mobile inverted bottleneck layer as the main building block as shown in Figure 3, which is an inverted residual block combined with squeeze-and-excitation (SE) blocks. An inverted residual block first projects an input feature map into a higher dimensional space and then applies depth-wise convolution operation in the new space. The new feature map is projected back to a low-dimensional space using point-wise convolution (1 × 1 convolution) with linear activation. Finally, a residual connection is added from the input to the output of the point-wise convolution operation, resulting in an output feature map. SE blocks, on the other hand, learn to weight channels of an input feature map adaptively. First, they convert the input to a feature vector of size equal to the number of channels (c) and then feed it to a two-layer neural network. The output of this network, which is a vector of size  c, is used to scale each channel based on its importance. Additionally, the baseline network is developed by leveraging a multi-objective neural architecture search technique that takes into account accuracy and real-world latency on mobile devices. Starting from this baseline network, the authors applied the compound scaling method to obtain seven different EfficientNet models (EfficientNet-B1 to B7).

### 3.3. Network Optimization on Ultrasound Images

It is a known fact that training deep neural network models such as EfficientNet requires having large labeled training examples, and collecting such data is time consuming and costly. An alternative remedy is to either use pre-trained models as off-the-shelf feature extractors and train a generic classifier (such as SVMs) or fine-tune the model for the classification problem at hand. Since we have a limited number of samples for the problem we are trying to address, we have choose to fine-tune the EfficientNet-B2 model for classifying LUS images.

### 3.4. Vison Transformers

Consider the collection of n chest medical images $S={\left\{X_{i},y_{i}\right\}}_{i=1}^{n}$, where $X_{i\;}\mathrm{and}\;y_{i}\;\mathrm{are}$ sample images and their associated class label, $\;y_{i}\in \left\{1,2,\ldots ,m\right\}$, and m is the number of identified classes for a set of images. The method’s goal is to learn how to translate the ultrasound image input to the appropriate class label. 

The prototype was inspired by a Vision Transformer (ViT). The vanilla Transformer [34], which has attracted a lot of attention recently for its capacity to deliver state-of-the-art (SOTA) performance in machine translation and other applications involving natural language processing [54], serves as the sole architectural foundation for ViT.

Encoder–decoder blocks of the Transformer design enable the concurrent processing of sequential data without the need for recurrent networks. The self-attention mechanism, which is suggested to capture long-range links between the sequence’s pieces, is substantially responsible for the success of Transformer models. In an effort to apply the standard Transformer to image categorization, Vision Transformer was proposed. Without incorporating any architecture tailored to certain types of data, the major objective was to generalize image categorization to modalities other than text. ViT performs classification by mapping a series of image patches to the semantic label using the encoder module of the Transformer in particular. Contrary to traditional CNN architectures, which frequently employ filters with local receptive fields, the Vision Transformer’s attention mechanism enables it to be applied across various regions of the image and to integrate data from throughout the entire image.

**Figure 4:**
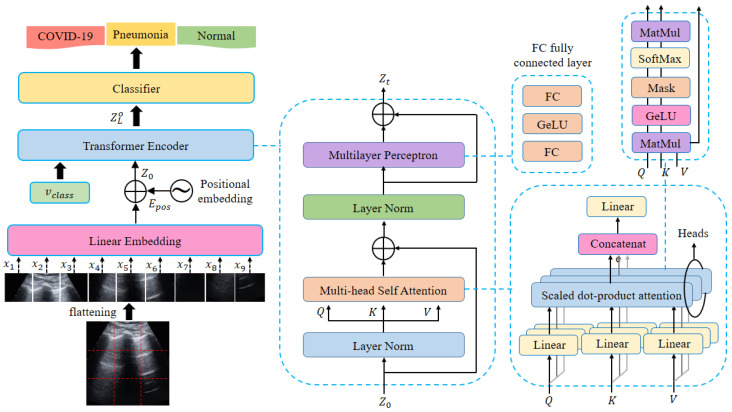
Vision Transformer Architecture.

Three key building components make up the proposed ViT model: an embedding layer, an encoder, and a final classifier. The input image is separated into non-overlapping patches in the first stage, which is then supplied into the embedding layer, encoder, and final classifier. We go into great detail about the model’s elements in the subsections that follow. The proposed ViT model’s general structure is shown in Figure 4.

#### 3.4.1. Linear Embedding Layer

First, a sequence of separate, non-overlapping patches are created from the input image. The input image x which has the dimensions h×w×c (where h, w, and c are the height, width, and number of channels, respectively) is then split into a series of lengths m by dividing it into small patches $x=\left\{x_{p}^{1},x_{p}^{2},\cdots ,x_{p}^{m}\right\}$, which have the dimensions p×p, which is fixed, and *m* is equal to $h\times w/p^{2}$. A typical patch size is 16 × 16 or 32 × 32; a smaller patch size yields a longer sequence, and vice versa.

The word tokens from the first Transformer are comparable to these patches. Using a learnt embedding matrix  E, the sequence of patches is linearly projected onto a vector of the model dimension *d* before being fed into the encoder. The learnable classification word $x_{class}$ that is necessary to complete the classification job is concatenated with the embedding representations after that. The flattened image patches are fed into a linear embedding layer E to match their dimension to the model dimension  d, and afterwards they are transformed into embeddings. 

Each patch embedding is added to its appropriate positional information to avoid the flattening operation from erasing the positional information. The learned class token $x_{class}$ is attached to the resulting position-aware embeddings. Through a self-attention process, the categorization token and patch embeddings communicate with one another.

(1) $$z_{0}=\left[x_{class};x_{p}^{1}E;x_{p}^{2}E;\ldots ;x_{p}^{m}E\right]+E_{pos}\;While\;E\in R^{\left(p^{2}\cdot c\right)\times d},\wedge E_{pos}\in R^{\left(m+1\right)\times d}$$

#### 3.4.2. ViT’s Encoder Module

The transformer encoder receives the resulting embedded patch sequence $\;z_{0}$. The encoder is made up of a stack of L identical layers, each of which is made up of two primary building blocks: a feed-forward network (FFN) block and a multi-head self-attention (MSA) block. The Transformer encoder’s main component, the MSA, uses the self-attention (SA) technique to identify dependencies between various patches of the input image. (2) and (3) both display specifics of the calculations that occur in the SA block. The input sequence is first used to create three distinct matrices: the key  K, the query  Q, and the value  V. An attention map is produced by using an inner product to match the query matrix to the key matrix. After being scaled by the key’s dimension $\;d_{K}$, the output is then obtained using the SoftMax function. In order to concentrate on more crucial parameters, the outcome is finally multiplied by the value  V.

(2) $$\left[Q,K,V\right]={\mathrm{zU}}_{QKV};U_{QKV}\in R^{d\times 3d_{K}}$$

(3) $$\mathrm{SA}\left(z\right)=\mathrm{softmax}\left({QK}^{T}/\sqrt{d_{K}}\right)\cdot V$$

Using multiple self-attention heads ($SA_{1},SA_{2}\ldots SA_{h}$), where *h* is the number of heads, the multi-head self-attention is an extension of SA that performs the SA procedure concurrently. The purpose of using h heads is to let each head to concentrate on a unique relationship between the image’s patches. Following that, a linear layer projects the outputs of all heads to the final dimension, as shown in Equation (4):

(4) $$\mathrm{MSA}\left(z\right)=\mathrm{Concat}\left({\mathrm{SA}}_{1}\left(z\right);{\mathrm{SA}}_{2}\left(z\right);\ldots {\mathrm{SA}}_{h}\left(z\right)\right)W^{O},W^{O}\in R^{h\cdot d_{K}\times d}$$

where $W^{O}$ stands for the final projection matrix’s learned parameters.

The MSA block is followed by the second block in the encoder layer, called FNN. It has a *GeLU* activation function [55] sandwiched between two completely linked layers. Each block of the two encoder layers is followed by a layer of normalization (LN). The outputs are calculated utilizing residual connections and the following Equations (5) and (6):

(5) $$z_{l}^{\prime }=\mathrm{MSA}\left(\ln\left(z_{l-1}\right)\right)+z_{l-1},l=1\ldots L$$

(6) $${\;z}_{l}=\mathrm{FNN}\left(\ln\left(z_{l}^{\prime }\right)\right)+z_{l}^{\prime },l=1\ldots L$$

The classification layer, which consists of a fully connected layer (FC) with a SoftMax activation function to generate the class labels, receives the output of the ViT encoder. We instruct the classifier to predict the class label using the classification token represented by the first element of the encoder output $\;z_{L}^{0}$.

(7) $$y=\mathrm{Softmax}\left(FC\left(z_{L}^{0}\right)\right)$$

### 3.5. gMLPs

**Figure 5:**
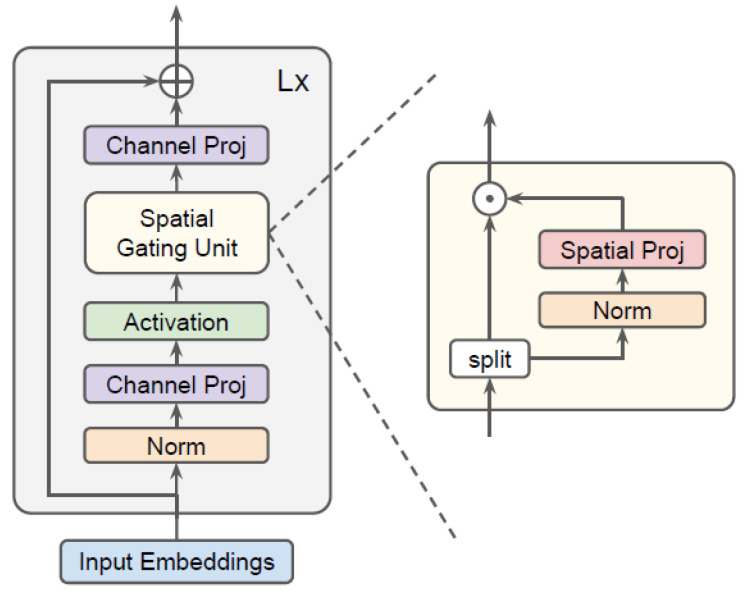
gMLP architecture with Spatial Gating Unit (SGU) [33].

Transformers’ multi-head self-attention layers are simplified by gMLP, which is suggested as evidence that self-attention is not essential for ViT [33]. gMLP is a simple variation of MLP with gating that includes static parameterized channel projections and spatial projections. As depicted in Figure 5, it consists of a stack of L identical blocks. The element-wise multiplication (linear gating) procedures known as linear projection operations ⊙ are used. BERT for NLP and ViT for vision are the input and output protocols. Positional encodings are not necessary for gMLPs, unlike Transformers, nor is there any need to mask out the paddings throughout the fine-tuning of NLP. 

## 4. Results

Given an ultrasound image of a patient, a multiclass model tells whether the patient has COVID-19 (has the coronavirus), is normal (does not have the coronavirus), or has pneumonia. As mentioned in the previous section, we applied transfer learning for this problem. To achieve this task, we fine-tuned three well-known architectures: EfficientNet-B2, ViT, and gMLP networks. Furthermore, we repeated the experiments without the transfer learning approach, i.e., learning from scratch. 

### 4.1. Dataset Preparation 

In order to conduct the experiments, we followed the same procedure followed by the author of the dataset [8]. They considered the recordings of convex probes and discarded the recordings of linear probes (20 videos and 6 images) and the recordings of viral pneumonia patients (6 videos). The convex videos vary in length and type and are composed of 160 ± 144 frames at a frame rate of 25 ± 10 Hz. These recordings (179 vides and 56 images) were manually processed and split into frames at a rate of 3 Hz (with 30 frames per video at maximum). At the end, the constructed ultrasound database contained images from three classes (i.e., 1204 COVID-19, 704 bacterial pneumonia, and 1326 healthy images). The irrelevant data (i.e., measure bars, texts, and artifacts on the borders) were cropped from the images before they were resized to 224 × 224 pixels. 

Similar to [8], all experiments in this study were repeated five times according to a 5-fold cross-validation procedure. In the 5-fold database split, a patient-based split was used, in which the images belonging to a single video are included in the same fold, and all three classes must appear in each fold. Applying the cross-validation method for database splitting is one of the known methods to evaluate the generalization capabilities and prevent overfitting in the predictive models [56,57]. 

### 4.2. Performance Evaluation 

As performance metrics, we report the accuracy (8), precision (9), recall (10), and *F*1 scores (11).

(8) $$Accuracy=\frac{TP+TN}{TP+FP+TN+FN}$$

(9) $$Precision=\frac{TP}{TP+FP}$$

(10) $$Recall=\frac{TP}{TP+FN}$$

(11) $$F1=\frac{2\times Precision\times Recall}{Precision+Recall\;}$$

where  TP,  TN,  FP, and  FN are the true positive, true negative, false positive, and false negative values, respectively. These evaluation metrics are used by several similar works in the literature [8,13,34,41]; thus, using them gives the ability to compare our results with those of the other studies and to be consistent with the assessment procedures of medical diagnostic systems [58]. These metrics are calculated from a confusion matrix. 

Accuracy is one of the main measures that could be used to analyze the performance of the classification systems. Moreover, precision and recall are two other performance measures that aid in discrimination between the classification systems. How many of the predictions are accurate is determined by precision, also known as positive prediction. On the other hand, recall (also known as sensitivity) demonstrates a system’s ability to identify a patient’s ailment. [58]. Recall shows how well the system is performing; false-negative predictions, or the inaccurate evaluation of infected patients as non-infected patients, come at a significant cost. Consequently, recall can be regarded as one of the most crucial criteria in the event of pandemics such as COVID-19. 

F-measure or *F*1 score is a well-known measure in the classification systems. It is calculated as the weighted harmonic mean of both precision and recall. *F*1 score is considered as an extra evaluation metric to differentiate between the systems that could generate a comparable value of precision and recall. 

These metrics’ equations are used for multiclass classification problems as in our problem by simply considering TP as the class we want to calculate metrics for and TN as the rest of the classes. 

### 4.3. Transfer Learning

As described earlier, we developed three models to detect COVID-19 through ultrasound imagery by applying a deep transfer learning approach. In this subsection, we report the results of the three proposed architectures.

#### 4.3.1. EfficientNet-B2

We fine-tuned EfficientNet-B2 for detecting COVID-19 using ultrasound images. We fine-tuned only the last 100 layers of this network and froze the remaining ones. We used the stochastic gradient descent (ADAM) for optimization with number of training epochs set to 20, mini-batch size set to 20, and learning rate set to 0.0001. Table 2 shows the detailed values of the different parameters of EfficientNet-B2, gMLP, and ViT networks.

**Figure 6:**
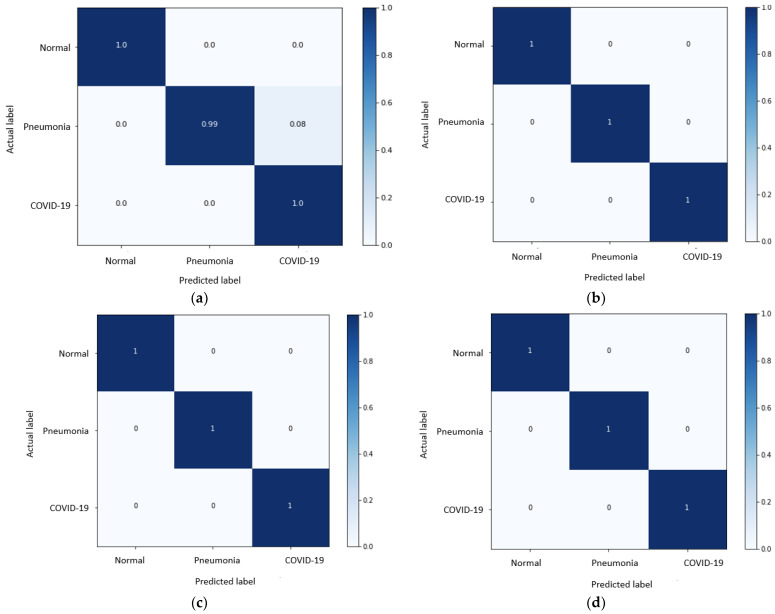

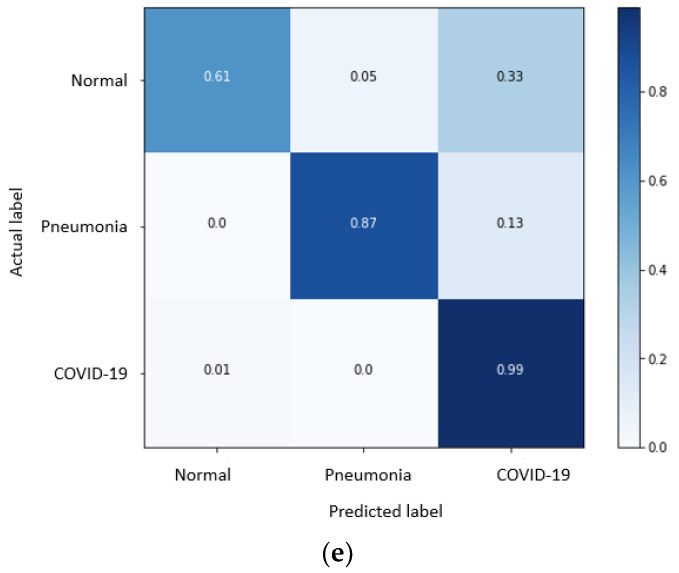
Confusion matrices of different 5-fold trials (**a**–**e**) of EfficientNet-B2. The rows represent the actual true values of the different classes; the columns represent the predicted values.

Figure 6 shows the confusion matrices of the different five trails of our experiments of the EfficicentNet-B2 model. We noticed that in five trials, COVID-19 class images were correctly classified at a percentage higher than 99.41%, leading to a high recall of 99.81%, which shows the ability of the proposed model to recognize COVID-19 images in most cases, even in the fifth trial, which shows low performance in classifying healthy and pneumonia classes. 

Starting from the confusion matrices in Figure 6, we calculated the accuracy of the EfficientNet-B2 model on the three classes (normal, pneumonia, and COVID-19) and present the results in Table 3.

The overall accuracy of the proposed architecture was 100% in three of the five trials and 99.83% in the first trial. The only exception is in the fifth trial, with an overall accuracy of 84.41%. It is worth noting that the accuracy of the system for detecting COVID-19 is excellent at 99.88% on average and 100% in four of the five trials.

The high accuracy of the architecture in detecting COVID-19 leads us to report the other evaluation metrics of detecting COVID-19 in Table 4.

Table 4 demonstrates that the proposed architecture exhibits high performance, with an average recall of 99.88%, precision of 95.84%, and *F*1 score of 97.41%. The excellent value for recall informs us that the system correctly classifies the images with COVID-19 markers.

#### 4.3.2. Contrasting EfficientNet-B2 with ViT and gMLP

In order to show the high performance of the proposed EfficientNet-B2 in classifying the ultrasound imagery, we now compare its accuracy in detecting COVID-19 with the results of two well-known deep learning techniques: gMLP and ViT16. The results are reported in Table 5. 

**Figure 7:**
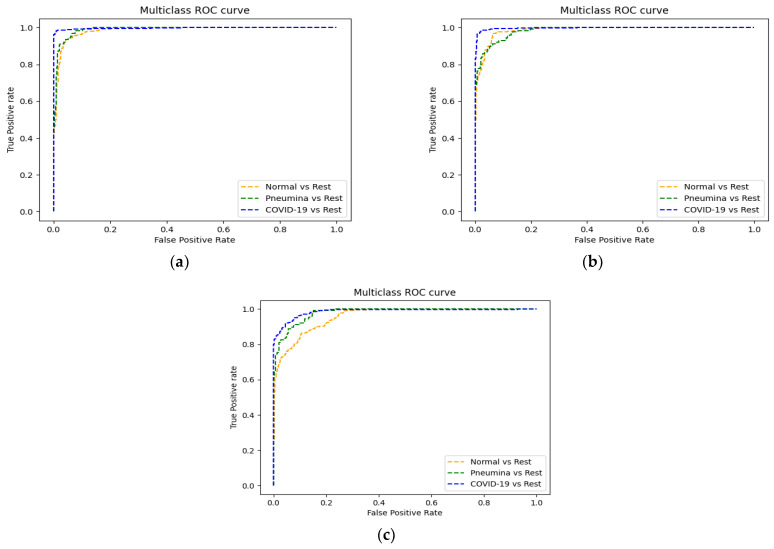
ROC curves of the three models: (**a**) EfficientNet-B2, (**b**) ViT, and (**c**) gMLP. We show the one-vs-rest ROC curve for the three classes.

In Figure 7, we show the receiver operating characteristic (ROC) curve with the average scores’ area under the curve (ROC-AUC) of the different classes by applying the three models. The results show that EfficientNets yields better results compared to ViT and gMLP. 

### 4.4. Learning from Scratch

In the previous sections, we explored the efficiency of the EfficientNet-B2 through transfer learning in classifying ultrasound images. Furthermore, we found that it outperforms gMLP and ViT. Therefore, in this section, we utilize EfficientNet-B2 to solve our problem with learning from scratch, i.e., without a transfer learning approach. Likewise, we compare the performance of EfficientNet-B2 with the performance of gMLP and ViT16 in terms of the accuracy of classifying COVID-19 class, as depicted in Table 6.

From the results in Table 6, we can also see that EfficientNet-B2 beats the other models with an average accuracy of 86.2% compared with accuracies of 77.48% and 70.96% for gMLP and ViT16, respectively.

### 4.5. Comparing with the State of the Art

In order to prove the superiority of our proposed models, we compare the results of our best model EffieceintNet-B2 with the models of the authors of the dataset as presented in Table 7.

From the reported results, we find that our proposed model achieves very good performance in terms of the evaluation metrics, with unbeatable improvement in all the reported evaluation metrics. 

### 4.6. Discussion

The reported results in the previous section show that the proposed approaches could clearly improve the performance of the COVID-19 detection systems using LUS. The results presented in Table 3 show that employing the transfer learning approach is a good choice to increase the accuracy of detecting COVID-19. Compared to the results of the learning from scratch approach shown in Table 6, transfer learning shows superiority and represents great improvement.

Furthermore, the proposed models based on transfer learning outperform the state-of-the-art methods on the same LUS dataset as shown in Table 7, where we can see that EfficientNet-B2 and even the other two models (gMLP and ViT) produce better results than the other works.

**Figure 8:**
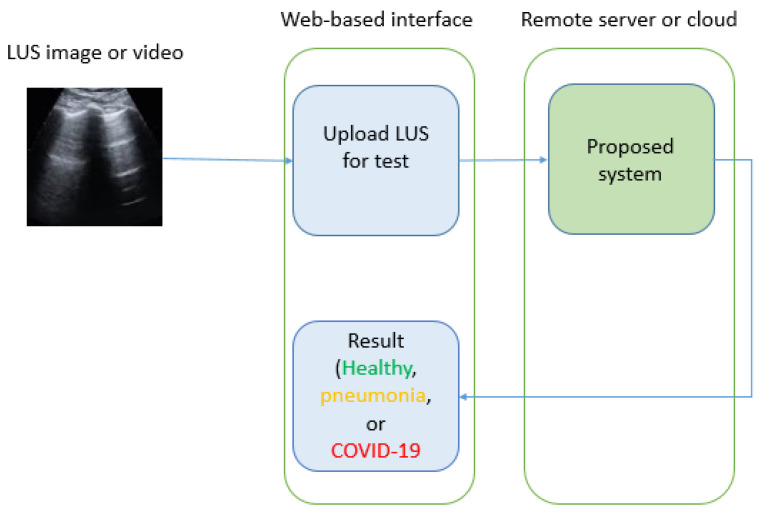
The deployment of the proposed model at the point of care.

In order to deploy the proposed model at the point of care, we can simply develop a web-based application that is connected to a remote server where the model operates. The technician at the point of care captures the LUS record and uploads it to the web-based application to send it for analysis. The model then receives the record and the frames are extracted, preprocessed, and classified. Finally, the result (the class of the LUS record) is sent back to the web-based application as shown in Figure 8.

The deployment of such a system has no bad effect on the patients or the medical teams and it is safe to be implemented and operated at the points of care. This is because ultrasound is considered a safe medical imagery technique and is cost effective. Furthermore, the only tools required are one ultrasound device and a computer at each point of care and one central computer for the model. 

## 5. Conclusions

In this work, we propose a deep learning approach to detect COVID-19 patients from ultrasound images. More specifically, we applied the transfer learning approach by fine-tuning a model of the well-known family of EfficientNet models, i.e., EfficientNet-B2. Moreover, we explored the performance of the model without using the transfer learning approach, i.e., learning from scratch. Furthermore, we contrasted the performance of EfficientNet-B2 with other well-known deep learning models (gMPL and ViT16) to classify LUS images. The experimental results for EfficientNet-B2 with transfer learning show exceptional performance, outperforming both gMPL and ViT16 models. EfficientNet-B2 shows acceptable performance even when applying it with a learning from scratch approach. Furthermore, our models outperform the models of the authors of the database, which demonstrates the high performance of our model. One of the limitations of our model (EfficientNet-B2) is the relative large number of parameters to learn. As for future work, we are planning to develop an automatic system for detecting COVID-19 from multimodal imagery, i.e., CT, CXR, and LUS. 

## Figures and Tables

**Table 1:** Number of videos and images in the dataset per class and probes.

	Convex		Linear		Total
	#Video	#Image	#Video	#Image	
COVID-19	64	18	6	4	92
Bacterial pneumonia	49	20	2	2	73
Viral pneumonia	3	–	3	–	6
Normal	66	15	9	–	90
Total	182	53	20	6	261

**Table 2:** Parameter optimization for EfficientNet-B2, gMLP, and ViT.

Parameter	EfficientNet-B2	gMLP	ViT
Number of iterations	25	25	25
Batch size	20	20	16
No. of classes	3	3	3
Input size	224 × 224	224 ×224	224 × 224
Optimizer	Adam	AdamW	Adam
No. of epochs	20	50	50
Learning rate	1 × 10^−4^	1 × 10^−4^	1 × 10^−4^

**Table 3:** Accuracy per class (%) of the EfficientNet-B2 model with a transfer learning approach. We present the results of the five trials, where each trial is an experiment of the 5-fold cross-validation procedure. The last column represents the overall accuracy of the model.

	Normal	Pneumonia	COVID-19	Overall Accuracy
Trial no. 1	100	99.20	100	99.83
Trial no. 2	100	100	100	100
Trial no. 3	100	100	100	100
Trial no. 4	100	100	100	100
Trial no. 5	61.44	87.30	99.41	84.41
Average ± std.	99.28 ± 19.27	97.30 ± 6.22	99.88 ± 0.29	96.79 ± 7.07

**Table 4:** Performance metrics (%) of the five trials of the EfficientNet-B2 model with a transfer learning approach; we report the results of COVID-19 class.

	Precision	Recall	F1
Trial no. 1	99.69	100	99.84
Trial no. 2	100	100	100
Trial no. 3	100	100	100
Trial no. 4	100	100	100
Trial no. 5	77.72	99.41	87.24
Average ± std.	95.84 ± 1.95	99.88 ± 0.29	97.41 ± 5.68

**Table 5:** Comparing the accuracy of EfficientNet-B2 in classifying COVID-19 class images with the accuracies of gMLP and ViT16.

	EfficientNet-B2	gMLP	ViT16
Trial no.1	99.83	91.51	90.7
Trial no.2	100	92.36	99.06
Trial no.3	100	98.62	97.64
Trial no.4	100	95.34	97.41
Trial no.5	84.41	83.58	80.38
Average ± std.	96.79 ± 7.07	92.82 ± 5.60	93.03 ± 7.78

**Table 6:** Accuracy of the proposed architecture by applying learning from scratch. The accuracy is compared with the accuracies of gMLP and ViT16.

	EfficientNet-B2	gMLP	ViT16
Trial no. 1	84.8	88.1	82.7
Trial no. 2	89.9	71.8	62.2
Trial no. 3	89.8	77.2	67.8
Trial no. 4	89.8	70.9	68.0
Trial no. 5	76.5	79.4	74.1
Average ± std.	86.2 ± 5.82	77.48 ± 6.93	70.96 ± 7.79

**Table 7:** Performance of the proposed architecture compared with the accuracies of models proposed by the authors of the dataset.

	Overall Accuracy %	Precision %	Recall %	F1 %
Born et al. [8]	87.8	90	88	89
Born et al. [12]	89	88	96	92
EfficientNetB2 (Transfer Learning) Proposed	96.79	95.84	99.88	97.41

## Data Availability

Not applicable.
